# Care Partner Perceptions of Conversation Difficulties, Strategy Use, and Psychosocial Impacts in Primary Progressive Aphasia: A Descriptive Study Using the PCI-DAT

**DOI:** 10.3390/brainsci16090938

**Published:** 2026-08-31

**Authors:** Angela C. Roberts, Matthew Bona, Emily Rogalski

**Affiliations:** 1 School of Communication Sciences and Disorders, Western University, London, ON N6G 1H1, Canada; 2 Department of Computer Science, Western University, London, ON N6G 1H1, Canada; 3 The Healthy Aging & Alzheimer’s Research Care (HAARC) Center, University of Chicago, Chicago, IL 60637, USA; mbona@bsd.uchicago.edu; 4 The Healthy Aging & Alzheimer’s Research Care (HAARC) Center, Department of Neurology, University of Chicago, Chicago, IL 60637, USA; emily.rogalski@bsd.uchicago.edu

**Keywords:** primary progressive aphasia, care partner, conversation difficulties, PCI-DAT, caregiving burden, communication strategies

## Abstract

**Highlights:**

**What are the main findings?**

- Communication partners of people with PPA report broad, heterogeneous conversation difficulties across subtypes.
- Perception of Conversation Index-Dementia Alzheimer’s Type (PCI-DAT) subscale scores did not differ significantly by PPA subtype (agrammatic, logopenic, or semantic).

**What are the implications of the main findings?**

- Care partner assessment and intervention planning in PPA should be individualized rather than subtype-driven.
- Item-level PCI-DAT data offer clinically actionable targets for communication strategy training.

**Abstract:**

Background: Primary progressive aphasia (PPA) disrupts communication and increases caregiving burden. Despite their critical role in supporting communication, objective data characterizing communication partners’ perceptions of conversation difficulties, strategies, and psychosocial impacts across PPA subtypes remain scarce. This study characterizes these experiences using the Perception of Conversation Index–Dementia of the Alzheimer’s Type (PCI-DAT) in a well-characterized PPA sample. Methods: Baseline PCI-DAT data from 95 communication partners of individuals with PPA enrolled in the Communication Bridge-2 (CB2) randomized controlled trial were analyzed. Descriptive analyses examined item- and subscale-level responses across five PCI-DAT subscales. Kruskal–Wallis tests were used to compare subscale total scores across agrammatic (PPA-G, n = 26), logopenic (PPA-L, n = 43), and semantic (PPA-S, n = 26) PPA subtypes. Results: Communication partners endorsed a wide range of conversation difficulties, with using the correct word and pausing/searching for words being the most frequently and severely rated items. Across subtypes, partners reported frequent use and perceived helpfulness of compensatory actions, both their own and those used by the person with PPA. PPA-S care partners reported directionally higher emotional burden (Subscale 4: M = 24.46, SD = 10.96) and practical challenges (Subscale 5: M = 19.27, SD = 11.61). No statistically significant between-subtype differences emerged for any subscale (all *p*-values > 0.12, ε^2^ = 0.030–0.044). Within-subtype variability was substantial across all subscales. Conclusions: These findings characterize the breadth and heterogeneity of communication partner-perceived conversation difficulties and their psychosocial impacts in mild-to-moderate PPA. Substantial within-subtype variability supports individualized assessment and intervention planning.

## 1. Introduction

Primary progressive aphasia (PPA) is a neurodegenerative dementia syndrome in which language decline is the first and primary presenting symptom [1,2]. Three research-defined subtypes differ in their language profiles: agrammatic variant PPA (PPA-G) primarily affects speech production and syntactic processing; logopenic variant PPA (PPA-L) is characterized by phonological working memory deficits and word retrieval difficulties; and semantic variant PPA (PPA-S) involves progressive loss of conceptual and semantic knowledge [1,3]. In some cases, individuals present with symptom profiles that do not conform strictly to a single subtype [3,4]. PPA has two main underlying neuropathological causes, with Alzheimer’s disease accounting for approximately 40% of cases and frontotemporal lobar degeneration (FTLD) pathologies accounting for 60% [4,5,6]. Average age at diagnosis is 65 years, and life expectancy after symptom onset is typically 7–10 years [4].

The language disruptions characteristic of PPA unfold within everyday relationships, most centrally affecting dyadic communication between the person with PPA and their primary care partner [7,8,9]. As PPA progresses, individuals become increasingly reliant on care partners to support communication, navigate conversation breakdowns, and sustain meaningful participation in daily life [10,11]. These increasing demands are associated with heightened emotional burden, shifts in relational dynamics, and diminished quality of life [7,12,13]. Importantly, the frequency and severity of conversation difficulties have been shown to correlate directly with both subjective emotional strain and objective caregiving burden in PPA [7].

Non-pharmacological interventions, including speech-language therapy, hold promise for reducing the severity and impact of language symptoms and supporting functional communication in PPA [14,15]. These include a growing focus on dyadic interventions that address communication challenges by modifying the behaviours of both the communication partner and the person with PPA [16,17,18,19]. The recently completed Communication Bridge-2 (CB2) randomized controlled trial (NCT03371706) is the largest global telehealth clinical trial of a speech-language intervention for PPA completed to date [16,17,20,21].

The CB2 trial demonstrated the efficacy of a telehealth-delivered, multicomponent, dyadic, and person-centered intervention in which both the individual with PPA and their primary communication partner were co-enrolled as active intervention participants [17]. The CB2 trial also incorporated personalized training stimuli and a web application for between-session practice [16,17]. Results showed that this approach was superior to a non-dyadic and impairment-focused intervention in achieving individualized goal attainment scaling (GAS) outcomes [17]. This expanded our findings from the Communication Bridge pilot [22], which showed that communication partner engagement levels significantly contributed to communication outcomes [23]. These findings underscore the importance of understanding care partner perspectives on conversation difficulties as a foundation for developing and evaluating targeted PPA interventions.

### 1.1. Importance of Communication Partner Perspectives in PPA

Contemporary approaches to PPA assessment increasingly recognize the importance of functional communication and communication partners perspectives alongside impairment-based language assessment [12,24,25,26,27,28,29]. Care partner-reported measures (CROM) offer a distinct perspective on the functional impacts of PPA that cannot be captured through clinician observation or language testing alone [30]. Previous research has demonstrated that communication breakdown is a major source of caregiver distress in PPA [7,13,31]. Carefully planned, personalized communication strategies can help care partners of people with dementia enhance everyday communication and mitigate the emotional and social consequences of caregiving [7,12,14,31]. A detailed characterization of care partner-perceived conversation difficulties and their impacts in PPA is therefore of direct clinical relevance for the design of person-centered interventions.

Despite this, a recognized gap exists in the availability of validated CROMs to capture conversation difficulties and their impacts in PPA [15,24,30]. One instrument with strong potential is the Perception of Conversation Index–Dementia of the Alzheimer’s Type (PCI-DAT), a 74-item CROM that captures care partners’ perceptions of conversation difficulties, communication strategies used by both members of the dyad, and the emotional and practical challenges associated with communication in dementia [32]. Originally developed and validated in Alzheimer’s dementia, the PCI-DAT has demonstrated good to excellent internal consistency (α = 0.79–0.97), robust test–retest reliability, and evidence of construct and concurrent validity in care partners of people living with Alzheimer’s dementia [32].

### 1.2. The PCI-DAT in the Context of PPA

Santos et al. (2026) provided the first evidence supporting the construct validity of the PCI-DAT in PPA [33]. Strong convergent validity was demonstrated for Subscale 1 (Conversation Difficulties) with the Communicative Effectiveness Index (r = −0.57, *p* < 0.0001), and for Subscales 4 (Feelings) and 5 (Challenges) with the Montgomery Burden Inventory Subjective Stress Burden scale (r = 0.59, *p* < 0.0001) and the Objective Burden Scale (r = 0.79, *p* < 0.0001), respectively [33].

Santos et al. identified a three-factor latent structure of the PCI-DAT in PPA through exploratory factor analysis, in which items from Subscales 2 and 3 coalesced into a Conversation Strategies factor and items from Subscales 4 and 5 merged into a Feelings and Challenges factor [33]. Overall, 87.8% of items showed good-to-excellent fit statistics in PPA [33]. Subscales 2 and 3, actions taken by the conversation partner and the person with PPA, respectively, were not assessed for convergent validity due to the lack of suitable comparison measures [33].

While this is an important foundational step, validation data alone do not characterize what communication partners experience across the five PCI-DAT domains, nor do they indicate whether perceptions differ across PPA subtypes. The current study addresses this gap by characterizing the frequency and severity of partner-perceived conversation difficulties (Subscale 1), communication partners’ reported use and perceived helpfulness of their own conversational actions (Subscale 2), communication partners’ perceptions of actions used by the person with PPA and their helpfulness (Subscale 3), and the emotional (Subscale 4) and practical (Subscale 5) impacts of conversation difficulties. Specifically, this study provides an objective, descriptive account of PCI-DAT responses from 95 communication partners of individuals with PPA enrolled in the CB2 trial, with exploratory between-subtype comparisons of subscale total scores. This work extends beyond the psychometric validation reported in Santos et al. [33] by characterizing the distribution and heterogeneity of communication partner experiences in PPA.

## 2. Materials and Methods

### 2.1. Study Design and Participants

All participants provided written informed consent. The study was conducted in accordance with procedures approved by the institutional review boards of Northwestern University and the University of Chicago.

This study presents a secondary descriptive analysis of baseline (pre-intervention) PCI-DAT data from the CB2 randomized controlled trial [17,21]. CB2 was a multi-site, telehealth-delivered, dyadic speech-language intervention trial that enrolled co-participating dyads comprising individuals with mild-to-moderate PPA and their primary communication partners. Communication partners were defined as adult family members or friends (aged 18 years or older) who were unpaid informal carers providing support with personal care, household management, health coordination, and communication and social support activities [33,34].

All participants were primary English speakers with adequate vision and hearing, whether aided or unaided, and sufficient technology skills to participate in a telehealth intervention delivered in English. Adequate vision and hearing were confirmed during telehealth screening using functional vision and hearing checks, including verification that participants could read and hear the presented material via video conference. Persons with PPA met published diagnostic criteria [1,2] as determined by a neurologist. They had mild-to-moderate PPA as determined by a PPA-validated telehealth version of the Western Aphasia Battery–Revised (WAB-R; Pearson, San Antonio, TX, USA) [35,36] with no more than mild apraxia of speech. Full inclusion and exclusion criteria are described in Roberts et al. [16] and Rogalski et al. [17].

### 2.2. Measures

The PCI-DAT was the primary measure of interest [32,33]. Subscale total scores reflect the sum of item ratings within each domain and are interpreted independently, given the differing anchor labels across subscales. The PCI-DAT consists of 74 items distributed across five sections: Subscale 1 (Conversation Difficulties) includes 22 items, Subscale 2 (Actions You Use) consists of 24 items, Subscale 3 (Actions Your Relative Uses) contains 12 items, Subscale 4 (Feelings) includes 8 items, and Subscale 5 (Challenges) includes 8 items. In the PCI-DAT, a response rating of 0 indicates that the participant does not experience or identify with the item. When present, behaviors, strategies, emotions, and challenges are rated on a Likert scale, with higher scores indicating greater severity, benefit, or impact. For Subscale 1, higher scores indicate greater frequency and severity of conversation difficulties (0 = never occurs; 7 = very serious problem). In Subscales 2 and 3, higher scores indicate the greater perceived benefit or effectiveness of the actions employed by the care partner or the person with dementia during a conversation (0 = does not do it; 7 = very helpful). In Subscales 4 and 5, higher scores indicate greater emotional impact and burdens on care partners related to conversation difficulties (0 = never occurs; 7 = very [impactful or burdensome]). In the original validation study [32] and in the lead author’s clinical experience with the PCI-DAT, it typically takes 10 to 20 min to complete.

Aphasia severity was characterized using the Western Aphasia Battery–Revised Aphasia Quotient (WAB-R AQ), a composite measure of overall language impairment, with lower scores indicating greater severity [35]. In CB-2, the WAB-R AQ was used as a global indicator of language severity alongside clinical assessment, rather than as a stand-alone method for diagnosing PPA or classifying its subtypes [16,21].

### 2.3. Data Acquisition

PCI-DAT instruments were mailed to participants. Communication partners completed the PCI-DAT independently and at their own pace and were not asked to record or report completion time. Written instructions were provided, and communication partners were encouraged to contact the research team with questions. Completed instruments were returned by mail and reviewed by a research team member for missing, ambiguous, or atypical responses, with follow-up contact as needed. Data were entered and verified in a CB2 trial REDCap database (Vanderbilt University, Nashville, TN, USA) hosted by Northwestern University [37,38] using double-entry procedures.

### 2.4. Analytic Approach

Missing data at the item level were exceptionally low, occurring in Subscale 1 (n = 5, 0.24%), Subscale 2 (n = 1, 0.04%), Subscale 3 (n = 4, 0.35%), and Subscales 4 and 5 (none). To preserve statistical power and prevent unnecessary loss of participant data due to listwise deletion, missing values were managed using item-level person-mean imputation.

Following standard psychometric recommendations, a subscale total score was calculated only if a participant completed at least 85% of the items within that specific subscale [39]. This threshold was met for all cases of missing data. Thus, the missing item response was replaced with the participant’s mean score across their completed items for that subscale, and the total score was recalculated using all items on the full scale. Given the negligible rate of missingness, this approach provides a reliable estimate of individual subscale totals without distorting the sample variance or introducing systematic bias [40,41].

To evaluate whether communication partners and persons with PPA differed across subtypes on baseline demographic and clinical characteristics, one-way analyses of variance (ANOVAs) were conducted for continuous variables (age, education, relationship duration, symptom duration, and WAB-AQ). For any omnibus test reaching statistical significance, post-hoc pairwise comparisons were conducted using Welch’s *t*-tests, which do not assume equal variances, with a Bonferroni correction applied across the three pairwise comparisons (corrected α = 0.0167). Categorical variables (communication partner sex, ethnicity, race, and relationship to the person with PPA) were evaluated using chi-square tests of independence when expected cell counts were sufficient (≥80% of cells with expected n ≥ 5).

Descriptive statistics were used to characterize PCI-DAT item-level and subscale-level responses. For each subscale, the percentage of communication partners endorsing each response category (0–7) is presented in a heat map table, with darker shading indicating a higher percentage of respondents. Subscale total score distributions by PPA subtype are displayed as violin plots.

The present descriptive analyses are organized according to the PCI-DAT’s original five-subscale structure [32]. This decision reflects two key considerations. First, latent factor structure and clinical subscale structure serve different purposes: the former describes statistical covariance among items and is appropriate for scale development and psychometric evaluation, while the latter preserves clinically interpretable domains with distinct anchor labels, item content, and intervention targets. The five-subscale framework in the original PCI-DAT is currently more familiar to clinicians using the instrument. Retaining it here ensures that the descriptive findings are directly interpretable within established clinical practice and intervention planning. Second, the three-factor solution identified by Santos et al. was derived from a single exploratory analysis and has not yet been replicated in an independent confirmatory cohort [33]. Thus, organizing clinical descriptive data around an unconfirmed latent structure would be premature.

To formally evaluate whether PCI-DAT subscale total scores differed across PPA subtypes, Kruskal–Wallis tests were conducted for each of the five subscales. Kruskal–Wallis tests were selected as an appropriate nonparametric approach given the ordinal nature of Likert-derived summed scores, the non-normal distributions observed within subtypes, and the zero-inflated item-response patterns characteristic of this instrument [42]. As described earlier, the PCI-DAT is a ‘semi-continuous’ Likert scale in which the absence of the behaviour/action/emotion is indicated by a ‘0,’ and the presence of a phenomenon is rated on a scale from 1 to 7. In the current dataset, this scale structure led to zero-inflated response patterns for select subscales [33].

Effect sizes were calculated as epsilon-squared (ε^2^), interpreted as small (0.01–0.05), medium (0.06–0.13), or large (≥0.14) [43]. A Bonferroni-corrected significance threshold of *p* < 0.01 was applied to account for multiple comparisons across five subscales. Dunn’s post hoc tests with Bonferroni correction (three pairwise comparisons per subscale) were planned for any omnibus result that reached statistical significance. Given that group sizes of n = 25–41 per subtype provide limited power to detect small effects, non-significant results are interpreted with this caveat in mind. All analyses were conducted using Python 3 (SciPy 1.17.1, NumPy 2.4.4, pandas 3.0.2).

## 3. Results

### 3.1. Participant Characteristics

Detailed demographic and clinical characteristics for the communication partners and persons with PPA are presented in Table 1.

The sample included communication partners of individuals with PPA-G (n = 26), PPA-L (n = 43), and PPA-S (n = 26). As a group, communication partners had a mean age of 64.7 years (SD = 10.2) and a mean education of 16.3 years (SD = 2.7). In the cohort, 85% of conversation partners were spouses. Persons with PPA had a mean age of 67.1 years (SD = 7.4) and a mean symptom duration of 3.7 years (SD = 1.8). Western Aphasia Battery Aphasia Quotient (WAB-AQ) scores across all three subtypes reflected mild-to-moderate aphasia severity (PPA-G: M = 83.38, SD = 6.47; PPA-L: M = 79.79, SD = 8.96; PPA-S: M = 82.03, SD = 8.84), consistent with the CB2 enrollment criteria [16].

PPA subtype groups did not differ significantly in communication partner age or sex, relationship duration, or person-with-PPA age, education, symptom duration, or WAB-AQ (all *p*-values ≥ 0.107). Communication partner education differed significantly across subtypes, F(2, 92) = 4.16, *p* = 0.019, η^2^ = 0.083. Post-hoc comparisons indicated that communication partners of persons with PPA-G had fewer years of education than those of persons with PPA-S (*p* = 0.005), whereas the other pairwise comparisons did not meet the Bonferroni-corrected significance threshold. Race, ethnicity, and relationship were not compared inferentially because of sparse cell counts.

### 3.2. Subscale Total Score Distributions by PPA Subtype

Descriptive statistics and Kruskal–Wallis test results for all five PCI-DAT subscales are presented in Table 2.

**Figure 1:**
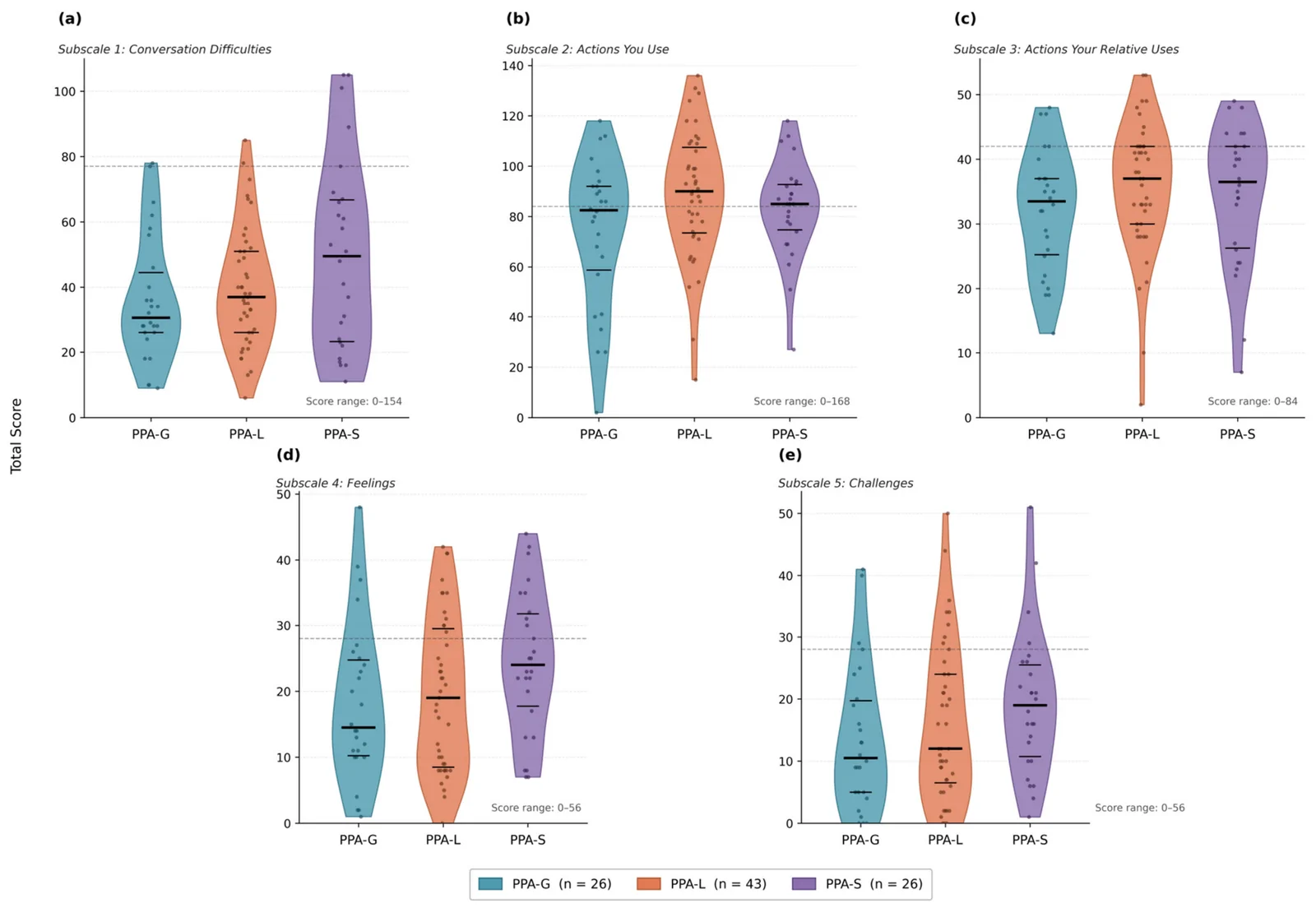
Distribution of PCI-DAT subscale total scores by PPA subtype. Panels (**a**–**e**) show score distributions for Subscales 1–5, respectively. The possible total-score ranges are Subscale 1, 0–154; Subscale 2, 0–168; Subscale 3, 0–84; and Subscales 4–5, 0–56. Violin plots display the full distribution of subscale total scores. Horizontal lines within each violin represent the 25th, 50th, and 75th percentiles with the median line bolded. Individual data points are shown as black dots. The dashed horizontal line in each panel indicates the scale midpoint (Subscale 1: 77; Subscale 2: 84; Subscale 3: 42; Subscales 4 and 5: 28), illustrating that scores across all subtypes fall predominantly in the lower half of the possible range. PPA-G = agrammatic variant PPA (n = 26; teal); PPA-L = logopenic variant PPA (n = 43; orange); PPA-S = semantic variant PPA (n = 26; purple). Kruskal–Wallis tests revealed no statistically significant differences across subtypes on any subscale (all *p*-values > 0.12; see Table 2).

Figure 1 displays the full distributions of subscale total scores across the three PPA subtypes.

A key finding shared across all five subscales is that the PPA subtype did not predict tightly clustered regions of scores. Substantial individual variability was observed within each subtype. No statistically significant differences across PPA subtypes were identified on any of the five subscales (all H < 4.15, all *p*-values > 0.12; Figure 1). Effect sizes were uniformly small (ε^2^ = 0.030–0.044), indicating that the PPA subtype accounts for less than 5% of the variance in subscale total scores across all five domains. As no omnibus test reached the Bonferroni-corrected significance threshold, Dunn’s post-hoc pairwise comparisons were not conducted.

Despite the absence of statistically significant differences across subtypes, consistent directional trends were observed. PPA-S communication partners had the highest mean scores on Subscale 1 (M = 49.65, SD = 29.09), Subscale 4 (M = 24.46, SD = 10.96), and Subscale 5 (M = 19.27, SD = 11.61), while PPA-G communication partners had the lowest means on all five subscales. For Subscale 2, PPA-L care partners reported the highest mean use and effectiveness of conversation partner strategies (M = 85.53, SD = 25.48). Subscale 3, the actions the person with PPA takes, showed the most comparable scores across subtypes (range of means: 31.27–35.74).

### 3.3. Subscale 1: Conversation Difficulties

Item-level percentage distributions for Subscale 1 are provided in Supplementary Table S1. Communication partners endorsed a wide variety of conversation difficulties. The most frequently and severely rated items were Q9 (has difficulty finding words) and Q10 (pauses or hesitates while searching for the right words), each receiving moderate-to-very-serious problem ratings (categories 4–7) from over 60% of care partners. In contrast, several difficulties were reported as not occurring by a substantial proportion of care partners, including making harsh or critical comments (Q12; 63%), having a short attention span (Q17; 49%), becoming anxious or frustrated when spoken to in a loud voice (Q16; 48%), orientation difficulties (Q18; 47%), and repeating the same idea or words (Q13; 44%).

### 3.4. Subscale 2: Actions You Use

Item-level percentage distributions for Subscale 2 are provided in Supplementary Table S2. Communication partners reported using a broad array of actions to support conversation at baseline. Remaining calm (Q17: 45% of respondents), filling in missing words (Q7: 27% of respondents), and repeating what was said (Q1: 27% of respondents) were rated as very helpful. Pretending to understand (Q16) was reported as not used by 63% of respondents and tuning out what the person says (Q21) by 76%, reflecting communication partners’ minimal use of avoidance strategies. Writing down what was said (Q2: 65% of respondents) and changing activity/redirecting (Q20: 55% of respondents), common dementia communication-support strategies, were rated as not used, with only a small proportion of participants rating them as very helpful (9% and 4%, respectively). This suggests that many CB2-enrolled communication partners were not using these strategies at baseline, despite their common recommendation as dementia communication supports. A different bimodal pattern was observed for talking more slowly (Q5): 32% of communication partners reported not using this strategy, while 23% rated it as very helpful, suggesting substantial variation in its use and perceived helpfulness across communication partners.

### 3.5. Subscale 3: Actions Your Relative Uses

Item-level percentage distributions for Subscale 3 are provided in Supplementary Table S3. Asking for help to find words (Q7: 32% of respondents) and searching for the right word (Q1: 21% of respondents) were perceived as very helpful by communication partners when employed by the person with PPA. Denying that there is a problem (Q9) was rated as not used by the person with PPA by 80% of care partners, and becoming withdrawn (Q10) by 45%, suggesting avoidance behaviors are less commonly observed or recognized by PPA care partners in this mild-to-moderate aphasia severity sample.

### 3.6. Subscale 4: Feelings

Item-level percentage distributions for Subscale 4 are provided in Supplementary Table S4. Frustration (Q1) and stress (Q3) showed the most distributed response patterns, with 37% and 30%, respectively, endorsing moderate-to-very-impactful ratings (categories 4–7). Anger (Q2) was rated as never occurring by 47% of care partners. Q8 (that you can accept the conversation as it is) showed a wide distribution, with 9% rating this as never occurring and 32% as moderately impactful in a positive way, reflecting substantial individual differences in communication partners’ adaptation to conversation difficulties.

### 3.7. Subscale 5: Challenges

Item-level percentage distributions for Subscale 5 are provided in Supplementary Table S5. Taking on new roles (Q1) and experiencing new burdens (Q2) showed distributed responses across the full scale. The inability to leave the person alone (Q6) was rated as never occurring by 71% of care partners, consistent with the mild-to-moderate PPA severity of this sample. Meaningful subgroups of communication partners endorsed moderate-to-high impact on loss of freedom (Q7: 51% never occurs, but 16% rated 4 or above) and loss of intimacy or closeness (Q8: 44% never occurs, but 23% rated 4 or above), underscoring that relational and personal consequences of conversation difficulties can be substantial for some communication partners even early in the disease course.

## 4. Discussion

Using the PCI-DAT, this study provides an objective, descriptive account of communication partner-perceived conversation difficulties, reported conversational actions and perceived helpfulness, and psychosocial impacts across a large, well-characterized sample of PPA communication partners, supplemented by formal between-subtype comparisons. Two key findings merit discussion: the breadth and heterogeneity of communication partner experiences within subtypes, and the absence of statistically significant between-subtype differences despite descriptive differences in mean scores.

### 4.1. Within-Subtype Variability Across PPA Subtypes

The most important finding from the Kruskal–Wallis analyses is that PPA subtype did not significantly predict total subscale scores on any of the five PCI-DAT subscales. Although the study was not powered to exclude small subtype effects, the small observed effect sizes and substantial overlap in score distributions suggest that subtype classification alone may have limited value for characterizing an individual communication partner’s experience. Considerable within-subtype variability was observed, reflecting differences among communication partners and dyads in conversational abilities and impacts. This finding suggests that the dominant source of variance is within-subtype individual variability, including differences in communication partners and dyadic differences in conversation abilities and impacts. This is consistent with the CB2 trial finding that subtypes did not cluster around particular outcome measure scores [17,20] and with the broader PPA literature, documenting high within-subtype variability even among individuals with clearly defined subtypes [6,44].

The considerable overlap in score distributions across subtypes suggests that subtype classification alone is insufficient to predict an individual communication partner’s burden profile. This pattern is consistent with evidence from the broader neurodegenerative disease literature. Beaton et al. examined caregiving burden using the Zarit Burden Interview across a large cross-disorder sample (N = 504) spanning Alzheimer’s disease/MCI, Parkinson’s disease, amyotrophic lateral sclerosis, frontotemporal dementia (FTD), and cerebrovascular disease [45]. They reported that although disorder-specific differences in caregiving burden were present, they were minimal. Within-group variability was substantial across all diagnostic categories, suggesting that diagnostic group membership is an insufficient basis for predicting individual caregiving burden [45].

Guger et al. reported a parallel pattern specifically within PPA and related frontotemporal lobar degeneration (FTLD) syndromes. Despite descriptively higher mean burden in PPA-S and behavioral variant frontotemporal dementia compared to PPA-G on the Zarit Burden Interview and Caregiver Strain Index, subtype differences did not reach statistical significance at baseline, and longitudinal burden trajectories correlated most strongly with neuropsychiatric symptoms, behavioral features, and caregiver age rather than with language-specific or cognitive severity measures [46].

Importantly, this pattern extends beyond burden measurement to intervention outcomes within the same CB2 cohort from which the current data are drawn. A planned follow-up analysis of the CB2 trial found no significant differences in responsiveness to the communication intervention across PPA subtypes when measured using Goal Attainment Scaling, demonstrating that subtype membership is a weak predictor of who benefits from dyadic, person-centred speech-language intervention [20].

Across burden measurement in broad neurodegenerative disease cohorts, caregiver strain in FTLD, and intervention responsiveness in PPA, diagnostic subtype may be insufficient on its own to characterize individual outcomes. The dominant source of variance lies within rather than between subtypes. In PPA specifically, individual-level factors such as disease duration, neuropsychiatric symptoms, the language impairment profile, care partner coping resources, relationship quality, and the person with PPA’s level of insight and care partner engagement may be important contributors to burden beyond subtype classification. These findings further support a shift toward person- and care partner-centered assessment approaches that characterize functional communication and individual communication needs beyond diagnostic subtype, consistent with emerging PPA assessment frameworks such as R.A.I.S.E. and other functional communication approaches [14,15,17,18,19,24,25,29,47].

### 4.2. Descriptive Patterns and Their Clinical Relevance

Response patterns across subtypes are of interest, although they should be interpreted with caution and warrant further study. PPA-S communication partners reported higher means on the Conversation Difficulties, Feelings, and Challenges subscales, although no significant subtype-specific differences in burden were observed in the present analyses. Future studies could determine whether semantic variant PPA imposes a dual burden on care partners.

The progressive loss of semantic knowledge in PPA-S can affect not only language but also social cognition, personality, and familiar conversational routines [46,48,49]. These clinical features may provide additional context for the observed descriptive pattern. Qualitative work in semantic dementia further illustrates how progressive changes in communication can extend beyond language impairment to alter shared activities, interpersonal roles, and the experience of maintaining a close relationship [48]. These relational consequences may contribute to the emotional and practical impacts reported by some communication partners in the present study. The observed co-occurrence of elevated emotional burden and impingement on communication partner activities in PPA-S is consistent with the Feelings and Challenges factor identified by Santos et al., in which Subscales 4 and 5 coalesced into a single latent dimension, suggesting that some care partners may experience emotional and practical impacts as a unified construct rather than as independent domains [33]. The importance of these psychosocial consequences is reflected in PPA clinical frameworks that emphasize counselling and communication-partner support as part of care, including attention to communication confidence, social engagement, and adaptation to progressive changes in communication [14,18,31,50].

The frequent use of supportive conversational actions and their perceived helpfulness across subtypes are also noteworthy. Proactive and supportive strategies, such as remaining calm, repeating what was said, and filling in missing words, were most often rated as helpful, whereas avoidance-oriented actions were infrequently endorsed. There are consistent patterns in actions rated as helpful by PPA communication partners in the CB2 trial and in prior studies of Alzheimer’s dementia caregiver-patient dyads [51,52], highlighting similarities across the broader spectrum of dementia disorders. However, this same literature highlights the potential for misalignment between care partners’ perceived and actual effectiveness of conversation strategies [52], underscoring that perceived helpfulness on the PCI-DAT in the current study should not be equated with observed clinical efficacy. The potential for misalignment between perceived and actual strategy effectiveness in the Alzheimer’s dementia literature also suggests that realigning care partners’ perceptions of strategy effectiveness may be an important intervention target, as explored in prior pilot studies [53,54]. However, this possibility requires replication in PPA before clinical application.

The descriptive convergence between communication partners’ own conversational actions and their perceptions of the actions used by the person with PPA mirrors the Conversation Strategies factor identified by Santos et al., in which Subscales 2 and 3 coalesced into a single latent dimension [33]. This reflects an orientation toward strategy use that aligns well with person-centered, dyadic, communication-focused PPA interventions [17,18,19,55]. The item-level heat map table distributions (S1–S5) provide a granular, clinically actionable picture of actions that communication partners reported using themselves or observed the person with PPA to use, as well as their perceived helpfulness. These findings may inform communication strategy training programs.

### 4.3. Characterization of Persons with PPA and CB2 Context

The CB2 trial enrolled individuals with mild-to-moderate PPA (WAB-AQ range of 55–98 across subtypes), an important contextual consideration when interpreting these findings. The level of aphasia severity in this sample may help explain the lower endorsement of severe conversation difficulties and the predominance of never-occurring ratings on some items (e.g., inability to leave the person alone, Q6: 71% never occurs). However, this period may also be an important opportunity for intervention as communication difficulties and care partner needs emerge. As PPA progresses beyond the mild-to-moderate stage, subtype differences in communication partner perceptions of conversation difficulties and their impacts, as well as the use and effectiveness of specific actions to mitigate conversation difficulties, may become more pronounced, a hypothesis that future longitudinal studies should examine. Given that the PCI-DAT was originally developed in Alzheimer’s dementia, future studies could also examine whether communication partner response patterns in individuals with PPA and evidence of underlying Alzheimer’s disease pathology or positive biomarkers converge with those observed in typical Alzheimer’s dementia as disease severity progresses.

Communication partner education differed across PPA subtypes, with lower educational attainment among communication partners of persons with PPA-G than PPA-S. Although no significant subtype differences were observed in PCI-DAT subscale scores, future studies with larger samples should consider communication partner characteristics as potential contributors to variability in perceptions of communication difficulties and their impacts.

### 4.4. Limitations

Several factors warrant consideration. Given sample sizes of n = 26–43 per subtype, non-significant results should therefore be interpreted as consistent with small or absent subtype effects rather than as definitive evidence of equivalence across PPA subtypes. Second, the sample was predominantly White, non-Hispanic, highly educated, and enrolled in a clinical trial that required a neurologist-confirmed diagnosis of PPA, which may limit generalizability to more diverse populations and those without access to expert care centers. Third, the study is restricted to mild-to-moderate PPA, and findings may not represent care partner experiences at more advanced disease stages. Fourth, PCI-DAT responses reflect communication partners’ ratings of their own actions and those of the person with PPA, representing their perceptions of effectiveness rather than direct clinical observation of dyadic behavior. Finally, item-level between-subtype analyses were not performed due to the considerable number of comparisons and the associated risk of Type I error; therefore, inferences regarding subtype differences at the individual-item level cannot be made.

## 5. Conclusions

This study provides the first detailed, objective, descriptive account of communication partner-perceived conversation difficulties, conversational actions and perceived helpfulness, and psychosocial impacts in PPA using the PCI-DAT. Communication partners of individuals with mild-to-moderate PPA reported a broad and heterogeneous range of conversational challenges, the use of diverse compensatory actions, perceived selected supportive actions as helpful, and emotional and practical burdens. No statistically significant differences across PPA subtypes were found on any PCI-DAT subscale, with small observed effect sizes and substantial overlap in score distributions. These findings underscore the importance of individualized, rather than subtype-driven, assessment and intervention planning in PPA. The findings may inform the development of targeted, dyadic communication interventions.

## Figures and Tables

**Table 1:** Baseline demographic and clinical characteristics of communication partners and participants with primary progressive aphasia (PPA) by PPA subtype.

	PPA-G (n = 26)			PPA-L (n = 43)			PPA-S (n = 26)		
Communication Partner Participants									
	M	SD	Range	M	SD	Range	M	SD	Range
Age at enrollment (years)	64.63	11.67	39–90	65.74	9.21	27–79	63.20	10.24	26–75
Education (years)	15.04	2.79	10–20	16.53	2.79	12–26	17.00	1.98	14–21
Relationship duration (years)	39.94	17.90	7–64	37.44	14.39	8–63	38.26	11.49	5–56
		**N**	**%**	**N**	**%**	**N**	**%**		
*Sex*									
Male		13	50	16	37	9	35		
*Ethnicity*									
Non-Hispanic		26	100	42	98	26	100		
*Race*									
White		26	100	41	96	26	100		
Asian		0	0	1	2	0	0		
*Relationship to person with PPA*									
Spouse		21	81	38	88	22	85		
Adult child		2	8	1	2	1	4		
Sibling		1	4	0	0	0	0		
Friend/neighbor		2	8	1	2	3	12		
Other		0	0	3	7	0	0		
**PPA Participants**									
	**M**	**SD**	**Range**	**M**	**SD**	**Range**	**M**	**SD**	**Range**
Age at enrollment (years)	66.50	7.13	53–80	68.55	7.46	52–82	65.39	7.34	53–79
Education (years)	15.65	2.54	12–20	16.53	2.25	12–21	17.04	2.42	12–21
Symptom duration (years)	3.48	1.69	1.6–8.3	4.00	1.77	1.3–8.6	3.59	2.00	0.4–8.4
WAB-AQ	83.38	6.47	63–97	79.79	8.96	55–98	82.03	8.84	60–95

Note. Values are reported as mean ± standard deviation (range) or frequency (N) and percentage (%). PPA-G = agrammatic variant PPA; PPA-L = logopenic variant PPA; PPA-S = semantic variant PPA; WAB-AQ = Western Aphasia Battery–Aphasia Quotient (higher scores indicate less severe aphasia; maximum = 100). Relationship duration = years of relationship with the person with PPA at baseline.

**Table 2:** PCI-DAT subscale total scores by PPA subtype and Kruskal–Wallis test results.

	PPA-G (n = 26)	PPA-L (n = 43)	PPA-S (n = 26)	H	*p*	ε^2^
	M (SD)	M (SD)	M (SD)			
Subscale 1: Conversation Difficulties	35.85 (19.11)	39.49 (18.42)	49.65 (29.09)	2.798	0.247	0.030
Subscale 2: Actions You Use	74.01 (29.78)	85.53 (25.48)	83.12 (19.44)	3.541	0.170	0.038
Subscale 3: Actions Your Relative Uses	31.27 (9.01)	35.74 (10.40)	34.42 (10.94)	2.800	0.247	0.030
Subscale 4: Feelings	18.15 (12.04)	19.58 (11.69)	24.46 (10.96)	4.144	0.126	0.044
Subscale 5: Challenges	13.58 (11.75)	15.98 (12.65)	19.27 (11.61)	3.818	0.148	0.041

Note. M = mean; SD = standard deviation; H = Kruskal–Wallis test statistic; *p* = *p*-value; ε^2^ = epsilon-squared effect size. Bonferroni-corrected significance threshold: *p* < 0.01. PPA-G = agrammatic variant PPA (n = 26); PPA-L = logopenic variant PPA (n = 43); PPA-S = semantic variant PPA (n = 26).

## Data Availability

The data presented in this study are available on request from the corresponding author (Emily Rogalski; erogalski@uchicago.edu) due to privacy and ethical restrictions.

## Supplementary Materials

The following supporting information can be downloaded at: https://www.mdpi.com/article/10.3390/brainsci16090938/s1, Table S1: Percentage of Responses by Item: Subscale 1—Conversation Difficulties. Table S2: Percentage of Responses by Item: Subscale 2—Actions You Use. Table S3: Percentage of Responses by Item: Subscale 3—Actions Your Relative Uses. Table S4: Percentage of Responses by Item: Subscale 4—Feelings. Table S5: Percentage of Responses by Item: Subscale 5—Challenges.
