# Supplementary material for: Care Partner Perceptions of Conversation Difficulties, Strategy Use, and Psychosocial Impacts in Primary Progressive Aphasia: A Descriptive Study Using the PCI-DAT
Source: Brain Sci. 2026 Aug 31;16(9):938. doi: 10.3390/brainsci16090938 (PMC13604639; doi:10.3390/brainsci16090938)
Supplement: Supplementary file 1 [file brainsci-16-00938-s001.zip › brainsci-4465377-supplementary.pdf]

Supplementary Material

Care Partner Perceptions of Conversation Difficulties, Strategy Use, and Psychosocial Impacts in Primary Progressive Aphasia: A Descriptive Study Using the PCI-DAT

Roberts, Angela; Bona, Matt; Rogalski, Emily
Correspondence: angela.roberts@uwo.ca

Contents

Tables S1–S5: Item-level percentage distributions for all five PCI-DAT subscales. Each table displays the percentage of care partners (N = 95) endorsing each response rating (0–7) for each item. Darker cell shading indicates a higher percentage of respondents, as shown in the shade legend below. Blank cells indicate 0% endorsement.

Shade legend:

0%	1–5%	6–15%	16–25%	26–35%	36–45%	46–55%	>55%

Note. Teal shading intensity reflects the percentage of respondents endorsing each rating. Text colour switches to white for cells with >35% to maintain readability.

Table S1. Percentage of Responses by Item: Subscale 1 – Conversation Difficulties

Item	0	1	2	3	4	5	6	7
Q1: Says nothing	37	19	20	15	8		1	
Q2: Uses words that do not go together to form a clear idea (e.g., uses vague terms)	11	27	21	16	18	5	1	1
Q3: Has difficulty keeping a conversation going	16	32	12	11	22	2	7	
Q4: Only talks about a few topics	40	19	22	6	9	1	2	1
Q5: Has difficulty with telephone conversations	14	22	13	14	23	9	4	1
Q6: Forgets the details when telling a story	18	29	20	9	16	7	1	
Q7: Forgets the details after hearing a story	15	32	17	15	13	6	2	1
Q8: Mixes up the details while telling a story	18	33	18	12	10	6	1	1
Q9: Has difficulty finding words (e.g., names of people, objects, places)	1	5	13	15	36	18	12	2
Q10: Pauses or hesitates while searching for the right word(s)		11	14	17	25	25	7	1
Q11: Does not finish sentences	17	23	17	14	15	11	2	1
Q12: Makes harsh or critical comments	63	16	8	2	7	2		
Q13: Repeats the same idea or word(s)	44	25	14	7	4	4	1	1
Q14: Forgets what he/she wants to say	24	33	16	14	7	4	2	
Q15: Becomes confused	33	38	14	7	3		5	
Q16: Becomes anxious or frustrated when spoken to in a loud voice	48	18	8	9	9	3	3	3
Q17: Has a short attention span	49	23	11	3	7	4	2	2
Q18: Has orientation difficulties (e.g., with person, place, or time)	47	27	8	8	2	4	3	1
Q19: Needs sentences to be repeated	18	32	23	14	11	1	2	
Q20: Has difficulty following directions	26	27	15	16	9	5	1	1
Q21: Has difficulty understanding questions	22	27	24	11	11	1	2	2
Q22: Has difficulty understanding what is said to him/her	23	33	20	11	9	1	2	1

Note. Anchor labels: 0 = Never occurs; 1 = Occurs, but not a problem; 4 = Moderate problem; 7 = Very serious problem. Values represent percentages of care partner respondents (N = 95). Blank cells = 0%. PCI-DAT = Perception of Conversation Index–Dementia of the Alzheimer's Type.

Table S2. Percentage of Responses by Item: Subscale 2 – Actions You Use

Item	0	1	2	3	4	5	6	7
Q1: Repeat what you said	11	3	7	19	15	18		27
Q2: Write down what you said	65	4	4	2	6	2	6	9
Q3: Use gestures	11	2	2	5	19	16	23	22
Q4: Simplify what you said	14	1	1	6	16	15	24	23
Q5: Talk slower	32	3	1	3	16	7	15	23
Q6: Talk louder	45	19	8	7	11	3	3	3
Q7: Fill in the missing word(s) or information	4	1	2	4	20	13	28	27
Q8: Give more information	5	2	2	4	20	18	26	22
Q9: Continue talking	27	15	20	4	23	3	5	2
Q10: Ask questions	8	1	3	9	20	18	19	21
Q11: Ask for clarification	7	1	1	6	24	17	21	22
Q12: Ask him/her to repeat what he/she said	18	1	9		18	19	21	14
Q13: Say, "I do not understand"	17	4	9	8	21	9	16	15
Q14: Give choices of what you think he/she means	8	1	3	6	16	20	29	16
Q15: Try to figure out what he/she means	9	1	2	8	9	21	28	20
Q16: Pretend to understand	63	19	11	3	1		1	2
Q17: Remain calm	6	1	2		12	7	26	45
Q18: Go along with what he/she is saying	29	11	5	12	19	12	11	2
Q19: Do things yourself	25	5	7	7	26	5	14	9
Q20: Change the activity (e.g., redirect to different topic(s) or task(s))	55	3	2	8	18	6	3	4
Q21: Tune out (e.g., ignore) what he/she says	76	13	2	4		2	2	1
Q22: Show what you mean	21	3	5	18	14	22	17	
Q23: Use gestures	33	2	5	5	20	11	14	11
Q24: Become emotional (e.g., angry, frustrated, sad, or aggressive)	49	46		1	1		1	1

Note. Anchor labels: 0 = Do not do it; 1 = Not helpful; 4 = Moderately helpful; 7 = Very helpful. Values represent percentages of care partner respondents (N = 95). Blank cells = 0%. PCI-DAT = Perception of Conversation Index–Dementia of the Alzheimer's Type.

Table S3. Percentage of Responses by Item: Subscale 3 – Actions Your Relative Uses

Item	0	1	2	3	4	5	6	7
Q1: Searches for the right word(s)	1	5	6	24	22	20		21
Q2: Repeats what he/she said	14	1	5	12	28	16	11	14
Q3: Says, "Forget about it"	39	34	6	12	3		2	3
Q4: Asks questions	14	4	5	24	15	15	23	
Q5: Corrects him/herself after using the wrong word(s)	11	2	1	4	21	16	24	20
Q6: Says directly that he/she is having difficulties	19	5	3	16	11		24	22
Q7: Asks for help to find word(s) (e.g., names of people, objects, or places)	8	1	1	14	12		33	32
Q8: Pretends to understand	47	22	14	10	5	1	1	
Q9: Denies that there is a problem	80	13	4	1			2	
Q10: Becomes withdrawn	45	37	11	5			2	
Q11: Becomes emotional (e.g., angry, frustrated, sad, or aggressive)	45	33	11	4		6		1
Q12: Looks confused	29	16	20	13		20		2

Note. Anchor labels: 0 = Do not do it; 1 = Not helpful; 4 = Moderately helpful; 7 = Very helpful. Values represent percentages of care partner respondents (N = 95). Blank cells = 0%. PCI-DAT = Perception of Conversation Index–Dementia of the Alzheimer's Type.

Table S4. Percentage of Responses by Item: Subscale 4 – Feelings

Item	0	1	2	3	4	5	6	7
Q1: Frustrated	7	29	11	14	22	12	3	2
Q2: Angry	47	24	14	4	8	1	1	1
Q3: Stressed	19	28	8	13	22	5	3	1
Q4: Sad	13	17	13	17	18	7	6	9
Q5: Lonely	45	16	8	3	13	3	7	4
Q6: A sense of emotional fatigue	27	22	4	11	17	9	7	2
Q7: A sense of loss	25	11	6	8	20	7	12	11
Q8: That you can accept the conversation as it is	9	5	6	4	32	16	15	13

Note. Anchor labels: 0 = Never occurs; 1 = Only a little; 4 = Moderately; 7 = Very. Values represent percentages of care partner respondents (N = 95). Blank cells = 0%. PCI-DAT = Perception of Conversation Index–Dementia of the Alzheimer's Type.

Table S5. Percentage of Responses by Item: Subscale 5 – Challenges

Item	0	1	2	3	4	5	6	7
Q1: You taking on new roles	9	22	5	14	23	12	8	6
Q2: New burdens for you	18	25	3	13	23	9	4	4
Q3: You having to anticipate his/her wants and needs	23	21	11	9	20	8	4	3
Q4: A change in the routine of your family life	23	26	7	11	17	6	6	3
Q5: A limit on your social activities	39	22	12	11	7	6	1	2
Q6: You not being able to leave him/her alone	71	13	4	2	3	3	1	3
Q7: A loss of your sense of freedom	51	20	4	8	8	6		2
Q8: A loss of intimacy or closeness	44	15	4	8	14	5	5	4

Note. Anchor labels: 0 = Never occurs; 1 = Only a little; 4 = Moderately; 7 = Very. Values represent percentages of care partner respondents (N = 95). Blank cells = 0%. PCI-DAT = Perception of Conversation Index–Dementia of the Alzheimer's Type.
